# Rowing Training in Breast Cancer Survivors: A Longitudinal Study of Physical Fitness

**DOI:** 10.3390/ijerph17144938

**Published:** 2020-07-09

**Authors:** Juan Gavala-González, Ismael Gálvez-Fernández, Pere Mercadé-Melé, José Carlos Fernández-García

**Affiliations:** 1 Department of Physical Education and Sports, University of Seville, 41013 Seville, Spain; jgavala@us.es; 2 Department of Didactics of Languages, Arts and Sport, University of Malaga, Andalucia-Tech, IBIMA, 29071 Malaga, Spain; jcfg@uma.es; 3 Department of Statistics and Econometrics, University of Malaga, Andalucia-Tech, 29071 Malaga, Spain; pmercade@uma.es

**Keywords:** breast cancer, rowing, fitness, anthropometry, heart rate

## Abstract

The aim of this study was to determine whether a rowing training program leads to improvements in physical fitness and body composition in women who have survived breast cancer (53.70 ± 7.88 years). The participants (n = 30) completed a twelve-week training program consisting of three sessions per week, with each session lasting from sixty to ninety minutes. An anthropometric and general physical fitness assessment was performed before and after the program. The results showed statistically significant improvements in all the physical fitness tests performed: sit and reach (2.82 cm); back scratch, dominant (3.29 cm); back scratch, non-dominant (4.59 cm); counter movement jump (1.91 cm); hand grip, dominant (2.54 kgf); hand grip, non-dominant (2.53 kgf); chair stand (2.56 rep); and six-minute walk (51.56 m). In addition, a significant improvement was observed in the efficiency of the cardiovascular system measured by heart rate, in beats per minute (bpm), both before (−12.63 bpm) and after the six-minute walk test (−11.46 bpm). The evaluated body composition parameters also improved, specifically total lean mass (2.18 kg) and the percentage of total body fat (−2.63%). We can therefore conclude that rowing training programs can be a strategy to be considered for improving physical fitness in this population given the improvement obtained in both anthropometric and physical fitness variables, thus leading to better health and quality of life.

## 1. Introduction

According to the International Agency for Research on Cancer (IARC), breast cancer is the most common cancer worldwide, affecting 2.1 million individuals each year and is the leading cause of death among women. In 2018, approximately 627,000 women lost their lives to breast cancer [1]. 

According to the 2018 IARC study, 4% of women worldwide will have breast cancer at some time in their lives [2]. This percentage is increasing, and it is expected that in 2040, the percentage of women who will be affected by this type of cancer will double, rising to 8% of the global population [3]. 

In contrast, breast cancer is no longer fatal but is becoming chronic, as the rate of survivors is above 70% five years after intervention. This percentage is associated with the effectiveness of treatments, to the increase in early diagnosis and to the prevention of relapse [4,5]. However, the risk of relapse 20–30 years after diagnosis is rather high [6]. The increase in life expectancy of these individuals is evidence that more people are living with the side effects of breast cancer treatment (loss of muscle mass and strength, loss of mobility and disability of the upper extremities, lymphedema, fatigue and cardiac toxicity) [7]. 

Physical activity decreases the duration and intensity of fatigue [8,9], reduces anxiety and depression [10], increases muscle mass and strength, decreases disability in the upper extremities [11] and improves quality of life [12,13], as well as restoring and even improving functionality and well-being in women who have overcome breast cancer [14]. Several meta-analyses and reviews affirm that physical activity also contributes to reducing the risk of breast cancer recurrence [15,16,17,18,19,20,21,22,23,24,25,26,27,28], while others have obtained neutral results, implying that physical activity does not cause any changes [29,30,31].

Currently, the literature provides no scientific evidence on the prescription and structure of physical exercise for breast cancer survivors [17,18]. Consequently, this study followed the recommendations of the American College of Sports Medicine (ACSM), which state that cancer survivors should perform 150 min of moderate aerobic activity or 75 min of vigorous aerobic activity per week and resistance training two days per week [32]. Physical exercise plays an important role in improving quality of life [11] and preventing or delaying the onset of other diseases in cancer survivors [15,16,17,18,19,20,21,22,23,24] by improving aerobic capacity and muscle strength [11] and reducing fatigue [8], all of which are key elements in recovery from this disease and its sequelae [32]. 

With regard to existing studies on physical activity and breast cancer, we highlight the study by Di Blasio et al. (2017) in which they carried out a strength program resulting in improved physical fitness [33]. The study by Keilani et al. (2016) implemented an aerobic program and found improvements in quality of life and physical fitness [34]. Studies that implemented mixed programs [35,36,37] reported improvements in physical fitness and body composition. Among them is the research by Dieli-Conwright et al. (2018), in which a combined 16-week aerobic and resistance program significantly improved fitness, with increases in both upper and lower extremity strength [35]. Similarly, Thomas et al. (2018) carried out a 12-month training program, finding a decrease in body fat (0.9%; 1 kg/m^2^) and an increase in lean mass (0.5 kg), as well as improved upper and lower extremity strength [36]. Modalities such as yoga and pilates also provide beneficial results in joint mobility, muscle strength and quality of life [20,23,27]. Finally, several studies on the effects of dragon boat training programs on breast cancer survivors have identified improvements in joint mobility ranges [38], strength in different body segments [38,39] and quality of life [40,41,42].

In our study, rowing was prescribed as the basis for a physical exercise program. This sport was chosen because it involves the muscles of both the lower and upper extremities [43] and almost all the muscles of the body [44], although the main feature differentiating rowing from most other sports is the cyclic and alternating action of the flexion and extension of the upper and lower limbs and the stabilizing muscles of the trunk and back during paddling to enhance technique [45]. While the existing literature on physical activity and breast cancer is scarce, in the case of rowing and breast cancer it is non-existent. For this reason, we believe that our study may represent an important advance in the prescription of healthy exercise for breast cancer survivors. Several studies have been conducted on dragon boat training and breast cancer [42,46,47,48,49], although a brief biomechanical analysis reveals that this is an asymmetric sport requiring compensatory effort and a series of difficult movements that are not recommended in this population. In contrast, rowing is a comprehensive sport that develops the lower, upper, front, back, right and left sides of the body in almost identical proportions, since the movement is symmetrical. At the same time, the position of the body segments is much more advisable for women who have suffered from cancer and surgery affecting the upper limbs, since rowing does not require forced movements. 

The aim of this study was to determine how a training program based on the sport of rowing may affect physical fitness and body composition in women who have survived breast cancer. 

## 2. Materials and Methods 

### 2.1. Design and Participants

The participants, all of whom were breast cancer survivors, and whose main characteristics are in Table 1, were recruited into a rowing club. Once selected, they met with the project coordinator, who explained the nature of the study, indicating that their anonymity would be maintained at all times, following the ethical considerations of Sport and Exercise Science Research [50] and in accordance with the principles included in the Declaration of Helsinki [51], which defines ethical guidelines for research on human subjects, and the University of Málaga gave the registered identification number for the ethics committee: 2020/REGSED-17007. All the participants signed a written informed consent form. In addition, during the entire intervention and thereafter, action was taken under the provisions of Organic Law 3/2018, of 5 December, on the Protection of Personal Data and the guarantee of digital rights regarding the protection of personal data in the Spanish legislation.

### 2.2. Instruments

Weight was measured to the nearest 0.1 kg using a Tanita model BC730 scale, following the protocol of the manufacturer for clothing and previous intake of liquids or food. Height was measured to the nearest 0.1 cm with a SECA model 213 portable stadiometer, according to the Frankfurt plane for body positioning. In the evaluation of body fat and muscle mass, a dual-energy X-ray densitometer (DXA, Hologic Explorer, Waltham, MA, USA) was used.

In addition, participants completed the physical fitness tests described below: 

The sit and reach test [52] is used as a general test of flexibility. The subject sits on the floor with knees straight, legs extended and with the soles of the feet pressed firmly against the box. The arms are extended forward with the palms facing downwards on the upper surface of the scale. In this position, the subject reaches forward as far as possible and holds the position. The score is the most distant point reached and held on the fourth movement. The test administrator stands close beside the scale and records the most distant line touched by the fingertips of both hands. If the hands reach unevenly, the hand reaching the shorter distance determines the score. The score is recorded to the nearest half inch. If the reach appears to be exactly half-way between two lines, the score is based on the last line actually touched. The best value of two observations was considered. The scale on the sit and reach box has an accuracy of 5 mm. 

The hand grip test [53] is used to measure the maximum isometric strength of the hand and forearm muscles. To start, the hand dimension is first measured, and then the grip area of the dynamometer is adjusted according to the length of the hand. Measurements were obtained under standardized conditions, with the participants in the seated position, elbow at ninety degrees and handle adjusted to the second position. After receiving an explanation of the procedures and becoming familiarized with the instrument, maximum grip strength should be applied for 3 to 5 s. The procedure was performed three times with each hand alternately, with an interval of one minute between each measurement. The highest value of three observations for each hand was considered. A TKK-5401 model digital hand dynamometer was used, which has a measuring range of 0.5 to 100 kg-force (kgf) and a minimum unit of measurement of 0.1 kgf. 

The counter movement jump [54] measures explosive and elastic force. It is performed with the subject starting from an upright position and with the hands on the hips. Then, an upward jump is performed by means of flexion followed as quickly as possible by extension of the legs. Knee flexion should reach an angle of ninety degrees and the trunk should be prevented from bending in order to eliminate any positive influence on the jump other than from the lower extremities. The legs during the flight phase should be extended and the feet at the moment of contact with the platform should be supported first by the metatarsal area and then by the back of the foot. Measurements were taken with the My Jump 2 mobile application [55]. 

The six-minute walk test [56] is a cardio-respiratory function test that measures the maximum distance a subject can walk for six minutes on a flat surface. The heart rate per minute is taken before and after the test. 

The chair stand test [57] assesses lower extremity strength by measuring the number of times the patient sits down and stands up from a chair, stabilized for safety. The subject sits in the center of the chair, with feet spread apart and resting on the floor. The arms are crossed and held close to the chest. From the sitting position, the subject stands up completely and then sits down again, repeating this cycle for 30 s. The final score is obtained by counting the total number of full squats (each time the subject stands up).

The back scratch test [58] evaluates flexibility in the upper extremities. The subject stands up and with the dominant hand must touch the shoulder of the same arm. With the fingers extended, the subject must advance along the back, bringing the elbow up until trying to reach the middle of the back. The other arm reaches around the back with the palm facing outwards, the goal being to touch the middle fingers of both hands behind the back. The distance between the tips of the middle fingers of both hands is measured. If the fingertips touch, the score is zero. If they do not touch, the score is negative, and if they overlap, the score is positive.

### 2.3. Procedure

Each participant, barefoot and wearing light clothing, underwent an initial anthropometric assessment of weight and height to calculate BMI. Subsequently, each participant performed the physical tests in the following order: hand grip, counter movement jump, back scratch, sit and reach, chair stand and six-minute walk. 

After the initial assessment, the participants completed a program of twelve consecutive weeks of rowing training (Table 2). For this program, each week included three days of training lasting between sixty and ninety minutes per session. These sessions were supervised by a personal trainer who ensured attendance, correct execution of the tasks and the intensity of the sessions, in addition to excluding from the study those participants who did not meet at least 90% participation. The training period was divided into three stages, with each stage lasting four weeks and each week having three sessions. The intensity and technical difficulty of these stages was progressively increased and was regulated through the subjective perception of the effort of the participants, using the Börg scale [59].

At the end of the twelve-week training program, the participants were re-evaluated using the same procedure used in the initial evaluation.

### 2.4. Data Analysis

The data were collected in an Excel spreadsheet, including age, height, weight, BMI, the results of the different tests and participant affiliations. Statistical analyses were performed using the Statistical Package for Social Sciences, version 25 (IBM Corp., New York, NY, USA).

To examine whether there were significant differences resulting from the rowing training, we analyzed the differences between the means of each variable pre- and post-training and then conducted parametric testing with Student’s *t*-test for related samples (paired data). Prior to this analysis, the normality of the distribution was verified through the Shapiro–Wilk test (*p*-value > 0.05 in all the variables under study). Regarding women’s age, 30.43% are aged 41 to 50, 52.17% from 51 to 60 years old and 17.39% from 61 to 70 years old. 

## 3. Results

Table 3 shows the means of the different variables both before and after training as well as their differences and comparisons to determine whether there are statistically significant differences. All the participants showed improvements in all the variables studied. In addition, these differences were all statistically significant except in the variables weight, BMI and total body fat, which although they improved after training, were not statistically significant: (diffWeight_Post-Pre_ = −0.37 ± 2.58; t = −0.798; *p* = 0.431; diffBMI_Post-Pre_ = −0.14 ± 1.02; t = −0.786; *p* = 0.438; diffTotal Body Fat_Post-Pre_ = −1.77 ± 7.6; t = −1.28; *p* = 0.211). The variables sit and reach (diff_Post-Pre_ = 2.82 ± 1.87; t = 8.24; *p* = 0.000); back scratch, dominant (diff_Post-Pre_ = 3.29 ± 3.15; t = 5.72; *p* = 0.000); back scratch, non-dominant (diff_Post-Pre_ = 4.59 ± 3.90; t = 6.44; *p* = 0.000); counter movement jump (diff_Post-Pre_ = 1.91 ± 1.71; t = 6.12; *p* = 0.000); hand grip, right (diff_Post-Pre_ = 2.54 ± 1.56; t = 8.93; *p* = 0.000); hand grip, left (diff_Post-Pre_ = 2.53 ± 1.91; t = 7.22; *p* = 0.000); chair stand (diff_Post-Pre_ = 2.56 ± 1.71; t = 8.19; *p* = 0.000); heart rate at the start of the 6-min walk test (diff_Post-Pre_ = −12.63 ± 14.68; t = −4.71; *p* = 0.000); heart rate at the end of the 6-min walk test (diff_Post-Pre_ = −11.46 ± 28.39; t = −2.21; *p* = 0.000); distance travelled in the 6-min walk test (diff_Post-Pre_ = 51.56 ± 48.26; t = 5.85; *p* = 0.000); total lean mass (diff_Post-Pre_ = 2.18 ± 4.81; t = 2.48; *p* = 0.019); and percentage of total body fat (diff_Post-Pre_ = −2.63 ± 5.43; t = −2.65; *p* = 0.013) all showed significantly positive differences between pre- and post-training. Practically all the variables that show statistically significant differences present the size of the effects, calculated from the Cohen’s d for a paired sample, being greater than 0.8. Therefore, the differences are statistically significant and clinically relevant.

In the following graphs, the variables can be observed grouped according to strength, aerobic, heart rate, flexibility and anthropometry. The graphs show how the variables have evolved before and after training and ratify the differences that have been analyzed in Table 2. 

**Figure 1:**
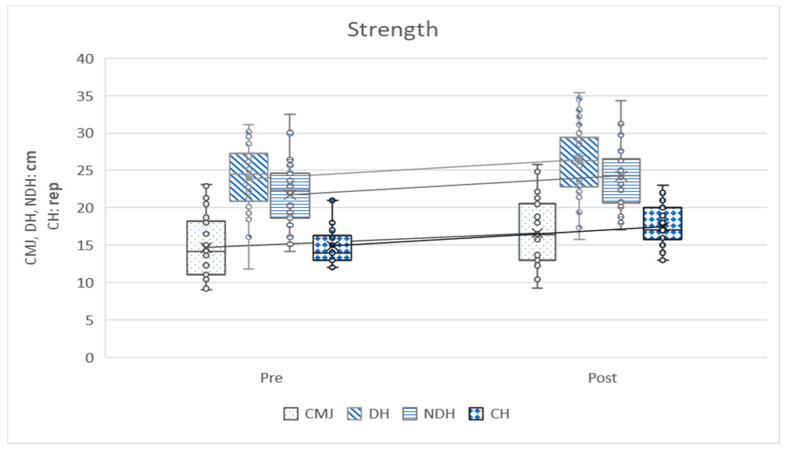
Strength variable. Pre = pretest; Post = post-test; CMJ = counter movement jump test; DH = dominant hand grip test; NDH = Non-dominant hand grip test; CH = chair stand test; rep = repetitions.

Figure 1 shows how all strength variables improve after training, with statistically significant differences. The chair stand test is the one with the greatest absolute difference (diffPost-Pre = 2.56 ± 1.71). 

**Figure 2:**
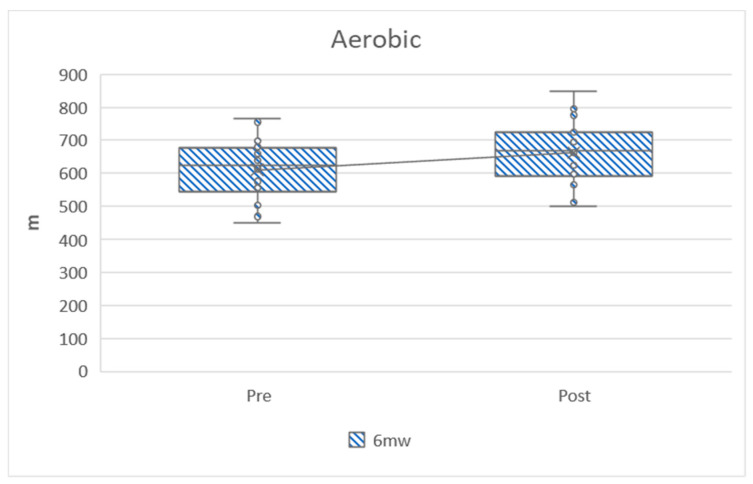
Distance in the six-minute walking test. Pre = pretest; Post = post-test; 6mw = six-minute walking test.

Figure 2 shows the aerobic variable, distance in the six-minute walking test, and a clear improvement is observed after training (diffPost-Pre = 51.56 ± 48.26).

**Figure 3:**
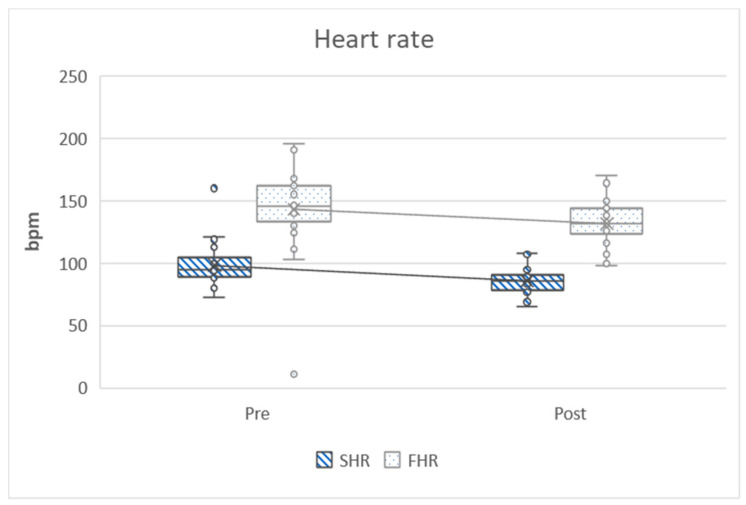
Heart rate in the six-minute walking test. Pre = pretest; Post = post-test; SHR = starting heart rate; FHR = final heart rate; bpm = beats per minute.

Figure 3 graphically analyzes the heart rate variables and clearly shows how these two variables decrease significantly due to training. 

**Figure 4:**
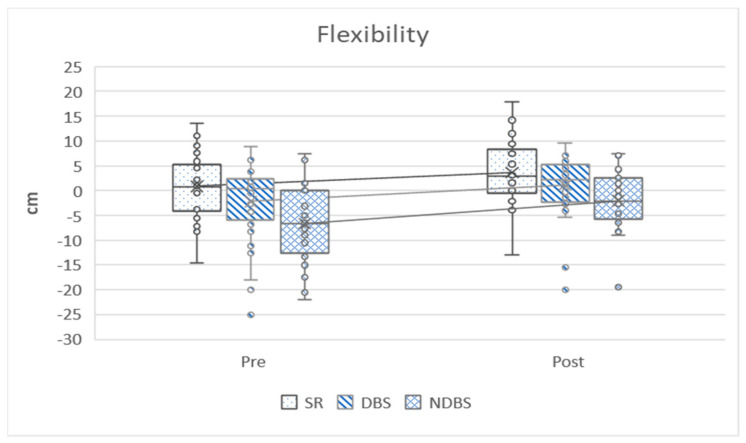
Flexibility variable. Pre = pretest; Post = post-test; SR = sit and reach test; DBS = dominant back scratch test; NDBS = non-dominant back scratch test.

Figure 4 graphically displays the flexibility variables where they all present better results after training, all of which are statistically significant.

**Figure 5:**
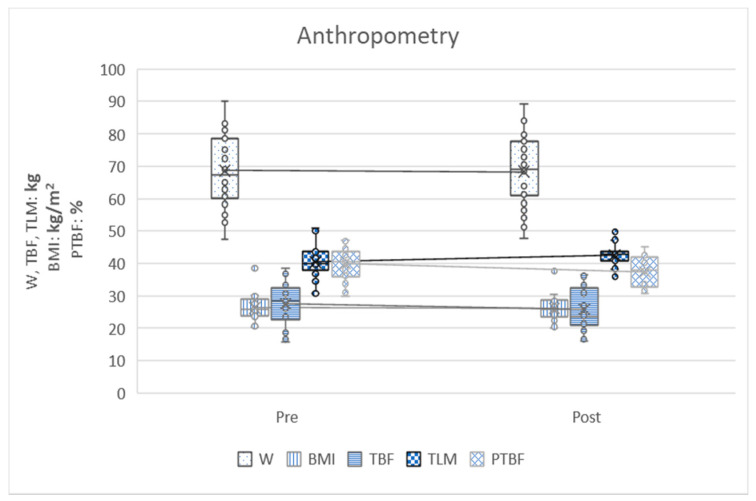
Anthropometry variable. Pre = pretest; Post = post-test; W = weight; BMI = body mass index; TBF = total body fat; TLM = total lean mass; TBFP = percentage of total body fat.

Figure 5 shows the anthropometric variables where all of them improve after the training tests. These differences are statistically significant.

To sum up, the results in Table 3 are displayed graphically in the five figures. All aerobic, strength and flexibility variables improve, since they present higher values after training. Heart rate variables improve by decreasing tensions at the end of the activity. Finally, the anthropometric variables improve by lowering the value of weight, body mass index, total body fat and percentage of total body fat, in addition to increasing the total lean mass once the rowing activity is finished.

## 4. Discussion

The study of breast cancer from the perspective of physical activity is relatively recent, as demonstrated by the limited literature and recommendations published by the ACSM [31] less than six months ago. A previous analysis identified only a small number of articles indicating that physical activity causes neither a positive nor a negative reaction in breast cancer survivors [29,30,31]. Most studies, however, have found that physical activity in individuals with breast cancer leads to a range of improvements, the most notable being a decrease in the perception of fatigue [8,9] and the delay or absence of other diseases such as breast cancer recurrence [15,16,17,18,19,20,21,22,23,24]. 

Physical activity has been shown to improve quality of life in breast cancer survivors [12,13] and functionality [14] in an isolated way or associated with aspects of physical fitness, including both joint mobility and muscle strength [20,23,27]. Through activities such as yoga or pilates [20,23], aerobic activities [32], specific strength programs [33], combined aerobic and muscle strength exercises [35,36,37] or the practice of dragon boat [42,46,47,48], improvements in physical fitness have been found, especially in strength in the extremities [36,37] as well as in body composition [35]. 

Several studies have conducted aerobic [33] or strength programs [34], which resulted in improvements in quality of life and physical fitness. However, it should be noted that most of the previous studies implemented mixed interventions following the recommendations of the ACSM [32]. In our rowing training program, the duration was twelve weeks, and the findings identified significant improvements in both the anthropometric and physical fitness variables analyzed. These results are in line with those of Dieli-Conwright et al. (2018) and Thomas et al. (2018), in which improvements in anthropometric variables were reported after a program of sixteen weeks and twelve months, respectively [35,36]. 

In our study, based on the administration of a rowing training program, improvements were observed in all body composition parameters evaluated through DXA, which decreased total body fat and percentage of total body fat and increased lean mass (Table 3). All of these improvements were significant and greater than in any previous studies in the literature. 

In addition, the results pertaining to physical fitness showed significant improvements in the different standardized tests that measure a wide range of manifestations of physical fitness including upper extremity and hip range of motion, lower and upper extremity strength, aerobic capacity and heart rate at rest and after prolonged effort. 

Consequently, the results of our study can contribute to improving the health and quality of life of women who have overcome breast cancer, offering them a new strategy for physical exercise prescription for a problem that affects more than two million women each year. 

## 5. Conclusions

Our research offers a novel methodological proposal for physical exercise prescription after breast cancer through a training program based on rowing, a cyclic and symmetrical sport that combines overall strength with aerobic endurance. This new proposal has a relatively short duration, twelve weeks, and is capable of bringing about improvements in all aspects of physical fitness and cardiac function during stress, as well as in anthropometric parameters such as fat and lean mass. 

## Figures and Tables

**Table 1:** Characteristics of the sample about breast cancer.

Age (Years)	Years from Diagnosis	Breast (%)		Stage (%)		Surgery (%)	
53.70 ± 7.88	6.57 ± 5.02	Right	26.09	I	4.35	Preservation	53.52
				II	30.43	Total Mastectomy	39.13
		Left	73.91	III	52.17		
				IV	8.7	Double Mastectomy	4.35

**Table 2:** Exercise prescription design for the program.

Stage	Content
1	Initial phase with mobility exercises, proprioceptive exercises and postural control exercises. Main phase with rowing training. Final phase with stretching. Börg scale 5–6.
2	Initial phase with mobility exercises, proprioceptive exercises and postural control exercises. Main phase with rowing training. Final phase with stretching. Börg scale 6–7.
3	Initial phase with mobility exercises, proprioceptive exercises and postural control exercises. Main phase with rowing training. Final phase with stretching. Börg scale 7–8.

**Table 3:** Statistical analysis of the studied variables. Mean ± standard deviation; differences, t-student test, effect size and p-value.

Variables	Pretest	Post-test	Diff Post-Pre	t-Student	Effect Size	*p*
Weight (kg)	68.67 ± 10.98	68.29 ± 10.79	−0.37 ± 2.58	−0.798	0.14	0.431
BMI (kg/m^2^)	26.34 ± 3.78	26.19 ± 3.67	−0.14 ± 1.02	−0.786	0.14	0.438
Total body fat (kg)	27.62 ± 6.79	25.85 ± 6.29	−1.77 ± 7.60	−1.28	0.23	0.211
Total lean mass (kg)	40.43 ± 4.61	42.61 ± 3.45	2.18 ± 4.81	2.486	0.45	0.019 *
Percentage of total body fat (%)	40.07 ± 4.84	37.44 ± 4.88	−2.63 ± 5.43	−2.657	0.48	0.013 *
Sit and reach test (cm)	0.87 ± 6.61	3.69 ± 6.57	2.82 ± 1.87	8.242	1.51	0.000 **
Dominant back scratch test (cm)	−2.19 ± 8.22	1.10 ± 6.54	3.29 ± 3.15	5.726	1.04	0.000 **
Non-dominant back scratch test (cm)	−6.64 ± 7.47	−2.05 ± 5.72	4.59 ± 3.90	6.44	1.18	0.000 **
Counter movement jump test (cm)	14.67 ± 4.30	16.58 ± 4.28	1.91 ± 1.71	6.128	1.12	0.000 **
Dominant hand grip test (kgf)	23.89 ± 4.56	26.44 ± 5.00	2.54 ± 1.56	8.933	1.63	0.000 **
Non-dominant hand grip test (kgf)	21.73 ± 4.37	24.26 ± 4.06	2.53 ± 1.91	7.229	1.32	0.000 **
Chair stand test (rep)	14.90 ± 2.41	17.46 ± 2.75	2.56 ± 1.71	8.194	1.50	0.000 **
Starting heart rate at six-minute walking test (bpm)	98.43 ± 16.72	85.80 ± 9.79	−12.63 ± 14.68	−4.712	0.86	0.000 **
Final heart rate at six-minute walking test (bpm)	143.26 ± 32.64	131.80 ± 18.62	−11.46 ± 28.39	−2.212	0.40	0.000 **
Distance in six-minute walking test (m)	611.23 ± 87.01	662.80 ± 85.82	51.56 ± 48.26	5.852	1.07	0.000 **

* = *p* < 0.05; ** = *p* < 0.001.
